# Understanding the determinants for predicting citizens’ travel mode change from private cars to public transport in China

**DOI:** 10.3389/fpsyg.2022.1007949

**Published:** 2022-10-10

**Authors:** Liming Sheng, Leibao Zhang

**Affiliations:** ^1^School of Public Finance and Taxation, Zhejiang University of Finance and Economics, Hangzhou, China; ^2^School of Business, Zhejiang University City College, Hangzhou, China

**Keywords:** travel mode change, private cars, public transport, norm activation model, theory of planned behavior, perceived accessibility

## Abstract

Rapid urbanization and motorization have generated increasing social and environmental challenges to the urban transport sector in China, such as traffic congestion, car accidents, air pollution, and global warming. Prioritizing the development of urban public transport system has been adopted as a primary strategy by Chinese government. However, the problems caused by large numbers of private cars are still far from being solved, and the ridership of public transport in China is relatively low. Therefore, the current study proposes a new comprehensive framework by enhancing the norm activation model (NAM) and theory of planned behavior (TPB) with the perceived accessibility to public transport, to better understand the determinants for predicting citizens’ choice of public transport as a sustainable travel mode. An online survey concerning travel mode change was conducted among citizens in Hangzhou, China. Based on 341 valid samples, partial least squares structural equation modeling (PLS-SEM) was employed to verify the proposed framework. The analytical results confirm that awareness of consequences and subjective norms are the two key constructs for connecting the two theories as a whole. Further, the examination of intention-behavior relationship shows that perceived accessibility to public transport could strengthen the relationship between behavioral intention and actual behavior in using public transport. The findings not only contribute to the development of pro-environmental theories, but also have meaningful implications for governments to develop relevant policies to encourage citizens to use public transport as a sustainable travel mode.

## 1. Introduction

The last few decades have witnessed the fast development of urbanization and motorization in China. However, the social and environmental problems accompanied with that have become an urgent need to be addressed by Chinese government, such as traffic congestion, car accidents, air pollution, and global warming. Compared with private cars, public transport is considered as a relatively sustainable travel mode due to its large capacity (Eboli and Mazzulla, 2015). In addition, travel mode change from private cars to other sustainable travel modes is considered to be more energy-efficient than methods of improving automotive technologies (Donald et al., 2014). Therefore, planning for public transport is a reliable solution to alleviate the social and environmental problems caused by the large number of private cars in urban areas.

Currently, Chinese government has prioritized the development of urban public transport system as a primary strategy to alleviate the problems caused by private cars. As a new first-tier city in China, the local government of Hangzhou has been investing huge resources to improve its public transport infrastructure, especially to develop its metro networks. However, the problems caused by the large number of private cars are still far from being solved at the moment, and the ridership of public transport in China is relatively low compared with that in Japan and Singapore (Zhang et al., 2019). On the other hand, previous research has reported that social and psychological motivations are more convincing for predicting an individual’s travel mode change than infrastructure differences (Hunecke et al., 2010). Therefore, improving infrastructure alone may not guarantee car reduction on urban roads, and it is necessary to further investigate the social and psychosocial motivations, which may predict the travel mode change from private cars to public transport.

In recent years, a growing body of literature has aimed to examine the social and psychosocial motivations in predicting public transport use. Passengers’ attitudes toward public transport services were found to be important in retaining current users and attracting new ones (Fu and Juan, 2017). Customer satisfaction surveys have been widely used to investigate passengers’ priorities of various service attributes of public transport, such as convenience, safety, comfort, flexibility, and reliability (Fellesson and Friman, 2008; Echaniz et al., 2018; Wang et al., 2020). Taking public transport as a product and improving public satisfaction could make it more attractive to the public indeed. However, the travel mode change from private cars to public transport is a complex behavioral change. In addition to the public’s satisfaction with public transport services, their attitudes toward private cars and public transport are also critical to affect travel mode change.

In previous studies, it has been found that driving a car could make the driver feel happy and represent one’s social status (Steg, 2005; Li et al., 2019), which may impede the travel mode change from private cars to public transport. However, feelings and attitudes toward private cars may vary from person to person. Previous studies have overlooked the negative aspects of driving on busy urban roads, such as constant traffic congestion, shortage of parking spaces, and unexpected car accidents, which could cause drivers’ negative emotions toward driving. Additionally, pro-environmental motivations may also help reduce private cars (Bamberg et al., 2007). Recently, Zhang et al. (2020b) found that awareness of the negative environmental consequences of using private cars could predict citizens’ intention to reduce car use. Nevertheless, studies concerning the negative aspects of driving that may lead to travel mode change are relatively rare in the existing literature. To fill this research gap, this study aims to explore the social and psychosocial motivations for travel mode change from two dimensions, i.e., the negative aspects of driving and the motivations of using public transport.

In the academic circles, two representative theories, namely the norm activation model (NAM) and the theory of planned behavior (TPB), have been widely applied to explain the behaviors of travel mode choice from the perspective of social psychology (Bamberg et al., 2007; Donald et al., 2014; Chen et al., 2019). In the existing literature, NAM was advocated by scholars who viewed travel mode choice as a pro-social behavior motivated by altruistic concerns (Bamberg et al., 2007), while TPB was popular among scholars who viewed travel mode choice as a behavior motivated by self-interest (Chen et al., 2019). However, little effort has been made to investigate the interrelationships among constructs from the two theories in the transport domain, and the understanding of travel mode change from private cars to sustainable transport modes remains surprisingly fragmented (Javaid et al., 2020). Therefore, this study aims to propose a new comprehensive framework with both altruistic concerns and self-interest, and incorporate NAM with TPB theories as a whole in pro-environmental researches.

In addition, few studies have explored the relationship between intention and behavior, because intention has been widely recognized as the main construct that leads to actual behavior in TPB. However, the declared intention is not identical variable to the actual behavior, and it has been found that an intention-behavior gap may exist in pro-environmental behaviors (Zhang et al., 2020a). In our previous work, it has been found that Hangzhou citizens are supportive of local public transport policies and have intention to reduce car use (Zhang et al., 2020b), but the problems caused by private cars are still far from being solved. Therefore, this study also contributes to the current literature and practice by further examining the intention-behavior relationship with the enhancement of an important domain construct, i.e., perceived accessibility (Lättman et al., 2016). By considering all the above arguments, the main focus and contribution of this study is to propose a new comprehensive framework from the perspective of social psychology by enhancing NAM and TPB with perceived accessibility to public transport, in order to explore the underlying determinants for predicting citizens’ travel mode change from private cars to public transport.

The remainder of this manuscript is structured as follows: Section 2 presents the related literature; section 3 illustrates the proposed framework; section 4 explains the main methodology applied in this study; section 5 describes the data analysis and tests the hypotheses; section 6 discusses the findings and provides a conclusion, as well as some implications.

## 2. Literature review and hypotheses

As mentioned in the last section, the understanding of travel mode change from private cars to sustainable transport modes remains fragmented, NAM and TPB are candidate theories in predicting sustainable travel behaviors with altruistic concerns and self-interest, respectively, and the intention-behavior relationship in the transport domain has seldom been explored. Therefore, attentions are given to the literature related to these two theories, their integration, and perceived accessibility, in order to provide a theoretical basis for the proposed research framework to predict sustainable travel behaviors.

### 2.1. Norm activation model

The NAM was first presented by Schwartz (1973, 1977) to analyze altruistic intentions and behaviors, such as donating bone marrow and helping others in daily life. There are three key constructs in the NAM, namely awareness of consequences (AC), ascription of responsibility (AR), and personal norms (PN). Specifically, AC refers to an individual’s awareness of undesirable consequences for others, AR indicates an individual’s perceived feeling of responsibility for undesirable consequences, and PN represents the perceived personal obligation to a certain action. The NAM suggests that AC is a guiding construct, which could either directly activate PN or indirectly influence PN through AR. Besides, both the direct and indirect effects of AC were confirmed for predicting citizens’ acceptance of green transport policies in China (Zhang et al., 2020b). The NAM has been successfully applied to predict pro-environmental intentions and behaviors in different research domains due to its excellent analytical capability (Zhang et al., 2018). For example, Werff and Steg (2015) adopted the NAM to explain residents’ energy-saving behaviors, and suggested that NAM constructs could predict multiple energy use behaviors in households, including food consumption, driving, and showering. Zhu et al. (2022) employed the NAM to explain tourists’ pro-social intentions during the pandemic period in order to promote sustainable tourism. The NAM has also been adopted to predict sustainable travel behaviors (Møller et al., 2018; Nordfjærn and Rundmo, 2019).

Therefore, based on the original NAM (Schwartz, 1973, 1977) and related works (Møller et al., 2018; Nordfjærn and Rundmo, 2019; Zhu et al., 2022), the following hypotheses (H1, H2, H3, and H4) are proposed from the perspective of altruism, i.e., taking travel mode choice as a pro-social behavior motivated by altruistic concerns.

H1: An individual’s awareness of consequences (AC) is expected to positively affect one’s personal norms (PN).

H2: An individual’s awareness of consequences (AC) is expected to positively affect one’s ascription of responsibility (AR).

H3: An individual’s awareness of responsibility (AR) is expected to positively affect one’s personal norms (PN).

H4: An individual’s personal norms (PN) is expected to positively affect one’s behavioral intention (IN).

### 2.2. Theory of planned behavior

Although some scholars have used the NAM as a primary research framework to study pro-environmental behaviors, including sustainable travel behaviors from the perspective of altruism, it is insufficient to regard the travel mode change as an altruistic behavior only. Instead, the travel mode change should be considered as a complex behavioral change arising from the combination of personal interests and the interests of others. Therefore, in addition to the NAM, theories based on self-interest, such as TPB, should also be explored in the research framework to understand citizens’ choice of public transport more comprehensively.

The TPB was first presented by Ajzen (1991), which has been used to predict pro-environmental intentions and behaviors in various research domains, including sustainable travel modes (Donald et al., 2014), organic food consumption (Canova et al., 2020), bicycle tourism (Lin et al., 2020), and waste separation practicing (Zhang et al., 2020a). According to TPB, there are three reliable antecedents that lead to behavioral intention, namely attitudes (AT), subjective norms (SN), and perceived behavioral control (PBC). Specifically, AT refers to a positive or negative personal feeling toward a certain behavior, SN represents a kind of perceived social pressure when performing or not performing a certain behavior, and PBC is defined as an individual’s perceived ability, either difficult or easy toward a certain behavior. Traditionally, TPB suggests that actual behavior (BE) is activated by behavioral IN, which is a reasonable decision-making process based on self-interest evaluation.

Some empirical studies have confirmed that the TPB framework is effective for predicting the public’s travel mode choice behavior. For instance, Donald et al. (2014) employed TPB to identify the determinants for predicting participants’ commuting travel mode and found that both car use and public transport use were influenced by TPB constructs among commuters. Similarly, Fu and Juan (2017) claimed that TPB constructs such as passenger attitudes and perceived behavioral control were crucial constructs to attract the public to use public transport, and further suggested that perceived behavioral control could directly lead to actual behavior.

Therefore, based on the original TPB (Ajzen, 1991; Donald et al., 2014), the following hypotheses (H5, H6, H7, and H8) are proposed from the perspective of self-interest, i.e., taking travel mode choice as an individual behavior motivated by self-interest.

H5: An individual’s perceived behavioral control (PBC) is expected to positively affect one’s behavioral intention (IN).

H6: An individual’s perceived behavioral control (PBC) is expected to positively affect one’s actual behavior (BE).

H7: An individual’s attitudes (AT) is expected to positively affect one’s behavioral intention (IN).

H8: Subjective norms (SN) is expected to positively affect one’s behavioral intention (IN).

### 2.3. Integration of the norm activation model and theory of planned behavior

From the afore-mentioned literature review, TPB could be applied to predict public’s decision-making process of travel mode choice. However, empirical studies have also suggested that the original TPB may overlook the individual’s moral aspect (Klöckner and Blöbaum, 2010; Si et al., 2020). In order to improve the predictive ability of TPB, Lo et al. (2016) incorporated PN from the NAM into the original TPB for predicting individuals’ commuting travel modes. Similarly, Si et al. (2020) selected and embedded two constructs from the NAM (i.e., moral obligation and AC) into the TPB framework to study the intention to use bike sharing, and claimed that perceived behavioral control and moral obligation were the two most prominent elements for predicting individuals’ intention to use sustainable travel modes. Although previous studies in the transport domain have tried to incorporate constructs from NAM to TPB, the interrelationships among NAM and TPB constructs in the transport domain have seldom been discussed.

On the other hand, Ajzen (2015), the original developer of TPB, suggested that the original TPB was an oversimplified representation, which made no limited assumptions about the veridicality of behavioral, normative, and control beliefs. This means that an individual’s attitudes, subjective norms, and perceived behavioral control could be induced by one’s beliefs from all perspectives. In the domain of green tourism, Han (2015) claimed that an individual’s beliefs about adverse consequences positively affect one’s attitudes and perceived behavioral control in choosing pro-environmental lodging. Similarly, Gkargkavouzi et al. (2019) also suggested that AC positively affects attitudes, subjective norms, and perceived behavioral control for predicting personal pro-environmental behaviors. In addition, the construct of subjective norms was confirmed to have a potential influence on PN in the existing literature on public transport use (Bamberg et al., 2007). Following previous studies, this study attempts to further investigate the interrelationships among constructs from the two theories in the transport domain.

Therefore, based on the literature review (Bamberg et al., 2007; Han, 2015; Gkargkavouzi et al., 2019) about the interrelationships among NAM and TPB constructs, the following hypotheses (H9, H10, H11, and H12) are proposed to integrate the two theories, i.e., NAM and TPB.

H9: An individual’s awareness of consequences (AC) is expected to positively affect one’s perceived behavioral control (PBC) toward driving.

H10: An individual’s awareness of consequences (AC) is expected to positively affect one’s attitudes (AT) toward public transport.

H11: An individual’s awareness of consequences (AC) is expected to positively affect subjective norms (SN) toward public transport.

H12: Subjective norms (SN) toward public transport is expected to positively affect an individual’s personal norms (PN) in travel mode change from private cars to public transport.

### 2.4. Perceived accessibility

According to the original TPB, individuals’ behavioral intention was the main antecedent directly leading to actual behavior. However, it has been argued that specific domain constructs are important to influence behavioral intentions and actual behaviors (Armitage and Conner, 2001; Donald et al., 2014). In the transport domain, accessibility is considered as an extremely valuable indicator to exert a positive influence on using public transport (Moniruzzaman and Paez, 2012; Cavallaro and Dianin, 2020). A generally accepted definition of accessibility is the potential opportunity for interaction (Hansen, 1959), which means the ease of reaching a destination by a particular travel mode (Dehghanmongabadi and Hoşkara, 2020). Although the concept of accessibility is easy to understand, it is limited in capturing personal subjective feelings of the same accessibility (Curl et al., 2015). In order to incorporate subjective feelings in evaluating accessibility in transport, Lättman et al. (2016) developed a new measurement called perceived accessibility. This refers to how easy it is to live a satisfactory life with the help of the public transport system (Lättman et al., 2016). Previous research in the transport domain has usually only focused on commuting for work, which may ignore an individual’s need for other social activities. In addition to commuting for work, travel for other social activities is also indispensable for publics, such as shopping, going to school, and meeting with friends and family members. However, limited focus has been placed on travel mode choice from the perspective of perceived accessibility. Therefore, the present study considers incorporating perceived accessibility into the proposed framework to strengthen the intention-behavior relationship in using public transport.

Therefore, based on the literature review (Armitage and Conner, 2001; Moniruzzaman and Paez, 2012; Donald et al., 2014; Cavallaro and Dianin, 2020) on the intention-behavior relationship with PA, the following hypotheses (H13, H14, and H15) are proposed to examine the intention-behavior relationship with the enhancement of the specific domain construct, i.e., perceived accessibility (PA).

H13: An individual’s perceived accessibility (PA) is expected to positively affect one’s behavioral intention (IN).

H14: An individual’s perceived accessibility (PA) is expected to positively affect one’s actual behavior (BE).

H15: An individual’s behavioral intention (IN) is expected to positively affect actual behavior (BE).

## 3. Framework

**FIGURE 1:**
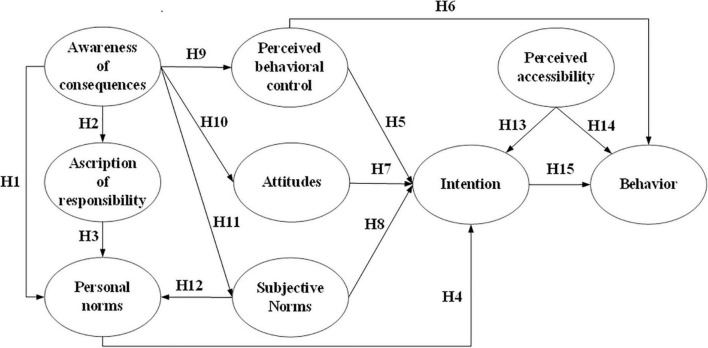
Modeling framework.

As shown in the literature review, NAM and TPB have been widely adopted by researchers to predict pro-environmental behaviors in various research fields, including behaviors related to sustainable travel mode choice (Klöckner and Blöbaum, 2010; Donald et al., 2014; Werff and Steg, 2015; Fu and Juan, 2017; Nordfjærn and Rundmo, 2019). Researchers have confirmed that each theory has the potential to predict behaviors from respective perspectives, i.e., altruism or self-interest. However, the interrelationships among the NAM and TPB constructs, as well as the intention-behavior relationship in the transport domain, have seldom been explored. To fill these research gaps, this study attempts to incorporate NAM and TPB theories, and enhance them with the specific domain construct, i.e., PA, to establish a new comprehensive framework to better understand citizens’ travel mode change from private cars to public transport, as shown in Figure 1.

## 4. Methodology

### 4.1. Data collection

In July and August 2020, an online survey concerning travel mode change was conducted among citizens in Hangzhou China using an online survey portal named Sojump^1^, which is known as one of the most professional online platforms for questionnaires and voting in China. Hangzhou was selected as the case study because it is a representative first-tier city with booming economy and heavy traffic in China. In addition, local government has invested heavily in public transport to alleviate traffic problems in recent years, and citizens are found to be supportive of local public transport policies (Zhang et al., 2020b).

The questionnaire was originally designed in English and translated into Chinese. To avoid a misunderstanding caused by language translation and guarantee the measurement scales are suitable for the specific society, two procedures were performed. First, a back-translation procedure was adopted before sampling. Second, small sample of volunteers were tested to check whether the statements of the questionnaire were clear and easy to understand. Based on the pretest results, the statements of the questionnaire were revised to ensure that the expression of the questionnaire could be fully understood by respondents.

The target population of this study was citizens who had been living in Hangzhou for at least 1 year, and were aged above the legal driving age (over 18 years old). The formal data collection was performed via online random sampling using China’s largest online survey portal Sojump, through which 367 Hangzhou citizens that were randomly selected from different residential areas answered the questionnaire. Specially, screening questions about age and duration of residence were set at the front of the questionnaire to exclude the non-target population. Following previous studies, the required sample size in this study was estimated by using the Power Analysis and Sample Size (PASS) software (Sapra, 2017; Zhang et al., 2020b). The confidence level could reach 99.9% with a sample size of 367 by setting in PASS with the urban district population size 6,353,000 in Hangzhou by the end of 2018 (Statistics Bureau of Hangzhou, 2019). Furthermore, a data cleaning procedure was performed to identify and remove invalid samples, i.e., missing data and outliers. Specifically, a total of 26 invalid samples including uncompleted samples and those who answered the questionnaire with dishonest attitudes were excluded, e.g., samples that finished the questionnaire in less than 1 min, answered all the questions with the same rating, or gave polarized ratings toward synonymous questions. Finally, 341 valid samples were retained. Although the retained 341 samples are a little less than the initial 367 samples, they meet the requirement of PLS-SEM guaranteeing reliable test results (Dirgahayani and Sutanto, 2020; Zhang et al., 2020b).

Common method bias (CMB) remains a potential problem in social studies, which refers to a systematic error caused by self-reported data obtained from the same source. Inspired by Al Halbusi et al. (2022), this study performed two procedures to reduce the potential CMB in data collection stage. First, a cover letter was used to inform respondents that their personal information and answers would be kept confidential. Second, differentiated scale options were adopted in demographic profiles. In addition, following the previous studies (Uzir et al., 2021; Alnoor et al., 2022), Harman’s single-factor test was also employed to exclude the potential problem of CMB. The test results in SPSS 22.0 showed that the largest variance of the single factor was 23.865%, which was less than 50% (Podsakoff et al., 2012), indicating that CMB was not a potential problem in this study.

Therefore, a total of 341 valid samples were obtained, and the demographic profiles of the valid respondents were in line with the Hangzhou Statistical Yearbook (Statistics Bureau of Hangzhou, 2019), as shown in Table 1. The proportion of male to female respondents is 42.82%/57.18%, which is close to the male-to-female ratio (49.47%/50.53%) in Hangzhou Statistical Yearbook. The age and income distribution all meet the spindle-shaped structure. In addition, a Chinese first-tier city is characterized of high level of car ownership and high level of education, which are consistent with Jia et al. (2018)’s work. In general, the samples are relatively representative for the urban population in Hangzhou.

**TABLE 1:** Demographic profiles.

Participants’ characteristics	Categories	Quantity (*n* = 341)	Percentage (%)
Gender	Male	146	42.82
	Female	195	57.18
Age	18–29	109	31.96
	30–44	160	46.92
	45–59	56	16.42
	60 and above	16	4.7
Driving license	Yes	263	77.13
	No	78	22.87
Car ownership	Yes	170	49.85
	No	171	50.15
Education level	Junior school or below	5	1.47
	High school	16	4.69
	College	220	64.51
	Master	88	25.81
	Doctor	12	3.52
Personal monthly income	Less than CNY2,000	34	9.97
	CNY2,001–5,000	72	21.11
	CNY5,000–10,000	131	38.42
	CNY10,001–20,000	64	18.77
	More than CNY20,000	40	11.73

### 4.2. Measurement design

To obtain data on citizens’ underlying motivations for travel model change, the questionnaire was divided into two parts, i.e., measurement of constructs and demographic profiles. Based on the proposed framework, the first part concerning items of measurement constructs was arranged into three sections in the questionnaire. Specifically, section 1 was related to NAM constructs, in order to analyze the travel mode behavior from the perspective of altruism. Section 2 was related to TPB constructs, in order to analyze the travel mode behavior from the perspective of self-interest. Section 3 was related to the PA constructs, in order to examine the intention-behavior relationship with the enhancement of the specific domain construct, i.e., PA. The second part about demographic profile was intended to collect basic personal information, such as age, gender occupation, travel mode, car ownership, and personal income. In addition, all the questions in the questionnaire were measured on a five-point Likert-type scale from “totally disagree” to “totally agree” except for the demographic profile. As shown in Supplementary Appendix Table 1, the items for measurement of constructs were adopted from previous research and revised to fit this study as follows.

In section 1, the participants were asked to express their degree of consent with reducing private car use, which were evaluated by questions on NAM constructs, i.e., AC, AR, and PN. These constructs have been proven to be capable of predicting citizens’ intention to reduce car use (Zhang et al., 2020b). Each construct for this section contained three items. The items for AC and AR were adopted from Steg et al. (2005), while those for PN were adapted from Ünal et al. (2019) to focus on the public transport mode.

In section 2, the participants were asked to express their AT toward public transport, the degree of consent toward SN, and PBC in difficulties of driving, behavioral IN, and actual BE in using public transport. Items for AT were adopted from Fu and Juan (2017) to evaluate public transport service satisfaction. Specifically, items for SN were adapted from Dirgahayani and Sutanto (2020) by adding two new items concerning social pressure from governments and social medias. Items for PBC were adapted from Kang et al. (2019) by extending the driving purpose from commuting for work to commuting for all daily activities. Items for IN and BE were adopted from Chen et al. (2019), which concerned pro-environmental travel behaviors. In section 3, the participants were asked to answer questions about perceived accessibility to public transport, which were adopted from Lättman et al. (2016). The perceived accessibility measure focused on evaluating the personal perception of opportunities that the participant could reach the destination to engage in daily social activities, such as going to school, commuting, shopping, and meeting with friends.

### 4.3. Structural equation model

Covariance-based structural equation modeling (CB-SEM) and partial least squares structural equation modeling (PLS-SEM) are two main approaches to structural equation modeling. Previous studies reported that PLS-SEM is superior to CB-SEM due to its robustness to collinearity and data distribution (Cassell and Bickmore, 2000; Uzir et al., 2021). To assess the proposed framework, this study employed the PLS-SEM approach, which has been widely used in behavioral research (Shen et al., 2016; Lopes et al., 2019; Bao et al., 2020; Abdelfattah et al., 2022; Naveed et al., 2022). The advantages of applying PLS-SEM approach in this study are as follows. First, as a promising statistical approach to the structural equation model, PLS-SEM can effectively assess a complicated model with multiple variables, whatever the variable is independent or dependent (Hair et al., 2017). Second, some unobserved psychological variables were included in the proposed model, which cannot be easily assessed using traditional statistical approaches (Hu et al., 2020). Fornell and Larcker (1981) suggested that unobserved variables can be measured with multiple measurement items using the SEM approach. Third, PLS-SEM is quite suitable for small sample sizes and complicated theories analysis (Hair et al., 2012). Therefore, this study employed the PLS-SEM approach to evaluate the measurement model and hypotheses with SmartPLS3.0 to test the collected data.

## 5. Results

In this section, the results of the data analysis are presented in two parts by using the PLS-SEM approach. First, the reliability and validity of the measurement model were checked with multiple criteria, such as factor loadings, Cronbach’s α, composite reliability (CR), and average variance extracted (AVE). Second, the relationships among constructs were evaluated with path coefficients and R^2^.

### 5.1. Measurement analysis

To evaluate the reliability and validity of the measurement model, a confirmatory factor analysis was performed using SmartPLS3.0. First, each item’s factor loading for a construct was evaluated. As suggested by Hair et al. (2019), the thumb rule is that the value of factor loading should be higher than 0.7, which suggests that the construct captures adequate variance of an item. Table 2 shows that all the factor loading values are higher than the benchmark of 0.7, ranging from 0.720 to 0.975, which indicates good content reliability in the constructs. In addition to factor loading, the AVE was applied to examine the convergent validity of items for a construct. As shown in Table 2, the AVE values range from 0.677 to 0.939 in this study, which are all above the recommended minimum value of 0.5 (Fornell and Larcker, 1981), indicating strong convergent validity of items for a construct.

**TABLE 2:** Reliability and validity.

Constructs	Factor loadings	Cronbach’s α	CR	AVE	R^2^
AC	0.850–0.889	0.842	0.905	0.760	
AR	0.882–0.941	0.904	0.940	0.839	
PN	0.898–0.932	0.898	0.937	0.831	0.418
PBC	0.813–0.853	0.778	0.871	0.693	
AT	0.905–0.944	0.920	0.949	0.862	
SN	0.720–0.894	0.880	0.913	0.677	
PA	0.813–0.934	0.904	0.933	0.778	
IN	0.916–0.948	0.929	0.955	0.875	0.733
BE	0.960–0.975	0.967	0.979	0.939	0.495

CR, composite reliability; AVE, average variance extracted; AC, awareness of consequences; AR, ascription of responsibility; PN, personal norms; PBC, perceived behavioral control; AT, attitudes; SN, subjective norms; PA, perceived accessibility; IN, intention; BE, behavior.

Second, Cronbach’s α and CR were used to evaluate each construct’s internal consistency reliability, and the recommended minimum value for both indicators is 0.70 (Fornell and Larcker, 1981; Hair et al., 2019). In this study, the threshold values of Cronbach’s α are between 0.778 and 0.967 as shown in Table 2, which exactly satisfy the recommended value of 0.7 (Cronbach, 1951; Hair et al., 2019). In addition to Cronbach’s α, CR has been suggested as a more reliable indicator for small sample size analysis (Hair et al., 2019). Table 2 shows that the threshold values of CR range from 0.871 to 0.979, which are much higher than the minimum criteria of 0.7 (Fornell and Larcker, 1981; Hair et al., 2019). Thus, the construct’s internal consistency reliability is successfully reconfirmed.

Third, discriminant validity was examined using the Fornell and Larcker criterion, in which the square root of each construct’s AVE is compared with each construct’s correlation coefficients with other constructs (Fornell and Larcker, 1981; Hair et al., 2019), as shown in Table 3. The bold values represent the value of square root of AVE for each construct, which is much higher than each construct’s correlation coefficients with other constructs, indicating suitable discriminant validity. Additionally, the cross-loadings of items could be used as an alternative measure to evaluate discriminant validity. As shown in Supplementary Appendix Table 2, the bold values representing each item’s factor loading to its own construct are all much higher than its cross-loadings to other constructs, which reconfirms the discriminant validity. Therefore, two approaches for evaluating discriminant validity ensured that every item surveyed in the questionnaire is exclusive to its own construct.

**TABLE 3:** Discriminant validity (Fornell and Larcker criterion).

Constructs	AC	AR	PN	PBC	AT	SN	PA	IN	BE
AC	**0.872**								
AR	0.405	**0.916**							
PN	0.464	0.446	**0.912**						
PBC	0.256	0.149	0.344	**0.832**					
AT	0.144	0.259	0.471	0.324	**0.928**				
SN	0.199	0.296	0.493	0.288	0.691	**0.823**			
PA	0.216	0.306	0.494	0.330	0.695	0.665	**0.882**		
IN	0.265	0.325	0.657	0.425	0.692	0.667	0.769	**0.936**	
BE	0.247	0.210	0.413	0.335	0.538	0.482	0.662	0.656	**0.969**

The bold values represent the square root of AVE. AC, awareness of consequences; AR, ascription of responsibility; PN, personal norms; PBC, perceived behavioral control; AT, attitudes; SN, subjective norms; PA, perceived accessibility; IN, intention; BE, behavior.

### 5.2. Hypotheses testing

To assess the effectiveness of the proposed model, hypotheses testing was conducted to explore the interrelationships among constructs after measurement analysis. Two indicators were evaluated in this section, namely the R^2^ and path coefficients.

First, the R^2^ is also known as the coefficient of determination, which captures the explanation ratio of an endogenous construct’s variance and is commonly used to measure the predictive power of a model in PLS-SEM studies (Shen et al., 2016; Lopes et al., 2019; Dirgahayani and Sutanto, 2020). As shown in Table 2, the values of R^2^ for the major endogenous constructs, i.e., PN, BE, and IN, are 0.418, 0.733, and 0.495, respectively. According to Cohen (1988), if the value of R^2^ for an endogenous construct is higher than 0.26, it could be considered as substantial in the field of behavioral science. Since the values of R^2^ for PN, IN, and BE are all above 0.26 in this study, it could suggest that the proposed model has a relatively strong explanation for predicting sustainable travel mode behaviors.

Second, the quantitative value and significance of the path coefficient were evaluated to verify the proposed causal hypotheses. The hypotheses are considered to be effective when the value of the path coefficient is higher than 0.1 (Hubert and Branden, 2003), and the P value lower than 0.05 suggests significance (Davison and Hinkley, 1997). As shown in Table 4, the path coefficients of H1 to H4 all meet the criterion of 0.1 and are significant at the 0.001 level, suggesting that the proposed hypotheses from the original NAM are both effective and significant. Similarly, as for the hypotheses from the original TPB framework, H5, H7, and H8 are all acceptable except H6. Since the path coefficient value of H6 is only 0.067 and its P value 0.189 exceeds the benchmark of 0.05, H6 should be rejected. The Hypotheses of H9 to H12 properly justify the interrelationships among constructs from NAM and TPB as the results for these hypotheses are both effective and significant. The hypotheses testing results of H13, H14, and H15 are all significant at the 0.001 level, presenting higher path coefficient values than other parts of the model, with values of 0.396, 0.385, and 0.332, respectively. Therefore, H13, H14, and H15 are also confirmed.

**TABLE 4:** Hypotheses testing results.

Paths	Sample mean	Standard deviation	*T* statistics	*P* values	Result
***NAM***					
H1: AC → PN	0.305	0.058	5.285	0.000	Accept
H2: AC → AR	0.408	0.051	7.869	0.000	Accept
H3: AR → PN	0.213	0.057	3.719	0.000	Accept
H4: PN → IN	0.288	0.044	6.548	0.000	Accept
***TPB***					
H5: PBC → IN	0.108	0.040	2.671	0.008	Accept
H6: PBC → BE	0.067	0.051	1.313	0.189	Reject
H7: AT → IN	0.165	0.057	2.847	0.004	Accept
H8: SN → IN	0.118	0.054	2.124	0.034	Accept
***Interrelationships among NAM and TPB constructs***					
H9: AC → PBC	0.259	0.059	4.318	0.000	Accept
H10: AC → AT	0.147	0.052	2.755	0.006	Accept
H11: AC → SN	0.203	0.051	3.888	0.000	Accept
H12: SN → PN	0.369	0.054	6.827	0.000	Accept
***Intention-behavior relationship with PA construct***					
H13: PA → IN	0.396	0.063	6.419	0.000	Accept
H14: PA → BE	0.385	0.071	5.404	0.000	Accept
H15: IN → BE	0.332	0.071	4.699	0.000	Accept

AC, awareness of consequences; AR, ascription of responsibility; PN, personal norms; PBC, perceived behavioral control; AT, attitudes; SN, subjective norms; PA, perceived accessibility; IN, intention; BE, behavior.

## 6. Discussion and conclusion

### 6.1. Discussion

In this part, the modeling results are described first, and commentaries are focused on the internal psychological process in terms of travel mode change.

According to the results of the hypotheses testing, the six main constructs from the original NAM and TPB, i.e., AC, AR, PN, PBC, AT, and SN, all contribute to the formation of behavioral IN to use public transport, but their contributions vary in patterns and sizes. Specially, PN, PBC, AT, and SN could directly impact on the IN, while AC and AR have indirect influence on IN through the afore-mentioned four constructs. From the perspective of influential power, PN from the NAM has the largest path coefficient (0.288) for predicting IN and the remaining three constructs (PBC, AT, and SN) from TPB have much weaker effects, with path coefficients (0.108 0.165, and 0.118) compared with PN.

In addition, two kinds of interrelationships among the NAM and TPB constructs are confirmed. First, the pro-environmental belief construct, i.e., AC from the NAM positively affects PBC, AT, and SN with path coefficients of 0.259, 0.147, and 0.203, respectively. This reveals that citizens who pay attention to the adverse consequences of using private cars may evoke a positive attitude toward public transport with strong pro-environmental society pressure, and generate perceived difficulties of using private cars on busy urban roads. Second, SN from the TPB positively affect PN, with a path coefficient of 0.369, which is much higher than the path coefficient of H8 (0.118) from SN to IN. This means that multiple social pressures from governments, social medias, friends, and relatives in encouraging sustainable travel modes could have a much stronger impact on shaping citizens’ moral obligation than behavioral intention to use public transport. This finding is slightly different from the previous study on the use of public transport conducted by Bamberg et al. (2007), who claimed that social norms directly influence PN but had no direct influence on intention. This difference might be caused by the additional items that were added in SN in this study. In this study, SN represents the social pressures not only from close friends or relatives, but also from the governments and social medias. Relatively strong predictive power of SN in this study might be attributed to the local collectivistic culture, where people may be more subject to influences from authorities compared with those living in a dominantly individualistic culture (Fu and Juan, 2017). Thus, this study contains more social information compared with the social norms construct in the study of Bamberg et al. (2007), which only focused on friends and relatives.

Furthermore, this study incorporates the domain construct of perceived accessibility to public transport into the proposed model, in order to improve the predictive ability of the proposed model and examine the intention-behavior relationship. The results of hypotheses testing for H13 and H14 confirm that PA to public transport positively affects behavioral IN and actual BE in using public transport, with path coefficients of 0.396 and 0.385, respectively. Meanwhile, IN positively affects BE, with a path coefficient of 0.332. These findings indicate that PA and IN may influence BE with similar sizes of path coefficients, which means that domain construct could play an important role in forming the actual pro-environmental behaviors (Armitage and Conner, 2001; Donald et al., 2014).

In this study, all the hypotheses in the proposed model have been proved to be effective except for H6, i.e., the path from PBC to actual BE. In addition, among the paths to IN, the path coefficient size from PBC was only 0.108, which is relatively weak compared with the influences from PN, AT, SN, and PA. This might be because the PBC construct is focused on evaluating the perceived difficulties of driving in this study, while previous studies usually examined the ease of using sustainable travel modes (Fu and Juan, 2017; Si et al., 2020).

### 6.2. Theoretical implications

The primary goal of this study is to propose a systematic framework to explore the underlying relationships among multiple variables so as to explain the citizens’ internal psychological process in terms of travel mode change. The results of this study have shown that constructs from NAM and TPB both have significant influences in shaping citizens’ behavioral intention to use public transport, which can be enhanced into a new comprehensive framework with the domain construct of perceived accessibility to public transport. This helps to understand the determinants for predicting citizens’ choice of public transport as a sustainable travel mode from a systematic point view.

First, it can be suggested that the willingness of travel mode change from private cars to public transport could be motivated not only by self-interest based on TPB, but also promoted by PN based on NAM. Differing from the previous studies, the present analysis also provides a deeper understanding for the interrelationships among NAM and TPB constructs by incorporating all the constructs from the two original models rather than involving a few selected constructs. Especially, this study confirmed that AC and subjective norms are two key constructs for building the interrelationships between NAM and TPB in terms of travel mode change.

Second, this study has confirmed that the declared intention is not identical variable to the actual behavior in terms of travel mode change, which is another important difference from previous studies in the area of travel mode change. The intention-behavior relationship is evaluated by including the domain construct of perceived accessibility, which means that actual behavior in sustainable travel modes is not determined solely by behavioral intention. In fact, the domain construct of perceived accessibility to public transport has much more influence power in forming actual behavior compared with intention. Therefore, the proposed framework could shed light on future works that the intention-behavior relationship should be taken into account in pro-environmental studies.

### 6.3. Policy implications

Based on the discussion above, some useful practical implications can be obtained to encourage citizens’ travel mode change from private cars to public transport.

The results of this study have confirmed that social and psychosocial motivations could predict citizens’ travel mode change from private cars to public transport, and give insights to policy makers that improving infrastructure alone may not guarantee car reduction on urban roads. In order to meet citizens’ daily travel needs, in-depth investigations of subjective feelings about different travel modes should be taken into consideration before planning or improving the public transport infrastructures.

Since PN, perceived behavioral control, attitudes, subjective norms, and perceived accessibility to public transport are all found to be influential in forming citizens’ intention to use public transport, governments should emphasize implementing policies that could promote pro-environmental subjective norms, enable citizens to feel obligated to reduce cars, and promote high perceived accessibility to public transport.

Particularly, subjective norms are considered as bridges in promoting governmental policies to citizens, which could deliver effective policy information and have a strong social impact on PN and the behavioral intention to use public transport. Additionally, social medias could also encourage citizens to use public transport. Local governments could cooperate with social medias to give campaigns on promoting new public transport facilities such as new subway lines, which could grab the attention of citizens who already have perceived difficulties in driving but hesitate to change their travel modes.

Last but not least, the perceived difficulties in driving might induce citizens to give up driving and choose to use alternative sustainable travel modes. However, this motivation does not seem strong enough to form actual behavior, because the perceived cost for driving may be still lower than that for taking public transport under certain circumstances. Thus, interventions from local governments are essential to deal with the problems in the urban transport domain. Push policies such as taxes on petrol and restrictions on car use in the city center are still necessary, which could increase driving cost. Meanwhile, pull policies such as subsidies on public transport and campaigns on promoting sustainable travel modes could increase citizens’ benefits from green travel behaviors, and could make public transport more attractive. As travel mode change is closely connected to everyone’s daily activities, it is suggested that perceived accessibility should be considered to meet citizens’ daily travel needs when governments make transport plans. For example, the public transport services providers could focus more on solving the last-mile problem (the distance from home to public transport facilities such as subways) by means of offering minibus routes and public bikes in order to improve citizens’ perceived accessibility.

## Conclusion

This study was inspired by the ongoing debate on solving the challenges caused by increasing private cars in the urban transport sector in China. It contributes to the existing literature by enhancing NAM and TPB with perceived accessibility in a comprehensive framework, and explores citizens’ motivations for the travel mode change from private cars to public transport. An online survey concerning travel mode change was conducted among citizens in Hangzhou China, then the PLS-SEM approach was used to test the measurement model and the proposed hypotheses. The analytical results reveal that perceived accessibility and behavioral intention are two important antecedents to form actual behavior in using public transport. Behavioral intention in using public transport is determined by both NAM and TPB constructs, among which AC and subjective norms are the two key constructs for connecting the two theories as a whole. In addition, the perceived behavioral control of car driving suggests that interventions from governments are necessary for forming actual sustainable travel behaviors. Overall, the considerably high values of R^2^ for PN, BE, and IN suggest that the proposed comprehensive framework has a strong predictive ability indeed, which could shed light on future studies concerning sustainable travel modes and other pro-environmental behaviors. Additionally, the results will also provide meaningful implications for governments to develop relevant policies to encourage citizens to use public transport instead of private cars. As travel mode change from private cars to public transport is a complicated behavioral process, the determinants may not be limited to the constructs in this work. The current work could be further improved by testing the influence of different personal characters, habits, and social cultures on travel mode choice behaviors in future studies.

Although this study explored the psychological process of sustainable travel mode decision-making based on NAM and TPB theories, it is still an exploratory study due to its relatively small sample size and simple random sampling technique. Future research direction could be cooperated with local governments or researchers from other countries in order to obtain more valid and general samples. Further, because the traffic issue is one of the major challenges facing all cities around the world, the framework proposed in this study could be used and examined in other countries to help to understand citizens’ decision-making process in sustainable travel mode profoundly.

## Data availability statement

The raw data supporting the conclusions of this article will be made available by the authors, without undue reservation.

## Author contributions

LS: data curation, investigation, and writing-original draft. LZ: project administration, supervision, and writing-review and editing. LS and LZ: conceptualization, formal analysis, methodology, contributed to the article, and approved the submitted version.

## Footnotes

1
https://www.wjx.cn
