# Supplementary material for: Understanding the determinants for predicting citizens’ travel mode change from private cars to public transport in China
Source: Front Psychol. 2022 Oct 10;13:1007949. doi: 10.3389/fpsyg.2022.1007949 (PMC9588939; doi:10.3389/fpsyg.2022.1007949)
Supplement: Supplementary file 1 [file Table_1.docx]

**Table A1.** Questionnaire items.

| **Constructs** | **Items** | **References** |
| --- | --- | --- |
| Awareness of consequences (AC) | AC1: Emissions from motor vehicles can lead to air pollution.  AC2: The exhaustion of fossil fuels is a social problem.  AC3: Global warming is a social problem. | (Steg et al., 2005) |
| Ascription of responsibility (AR) | AR1: I feel jointly responsible for the air pollution.  AR2: I feel jointly responsible for the energy problems.  AR3: I feel jointly responsible for the global warming. | (Steg et al., 2005) |
| Personal norms (PN) | PN1: I feel obliged to use a car as little as possible in my daily behavior.  PN2: I would be a better person if I protect our environment by choosing other means of transport rather than car.  PN3: I feel morally obliged to use public transport instead of car, regardless of what other people do. | (Ünal et al., 2019) |
| Perceived behavior control (PBC) | PBC1: It’s difficult for me to avoid traffic jams when driving out.  PBC2: It’s difficult for me to avoid using brake frequently when driving out.  PBC3: It’s difficult for me to speed up when driving home from work. | (Kang et al., 2019) |
| Attitudes (AT) | AT1: I feel convenient to travel by public transport.  AT2: I feel comfortable to travel by public transport.  AT3: I feel happy to travel by public transport. | (Fu and Juan, 2017) |
| Subjective norms (SN) | SN1: Social medias encourage me to use public transport.  SN2: Government transport policies encourage me to use public transport.  SN3: My family members and close friends like travelling by public transport.  SN4: My family members and close friends think I should use public transport.  SN5: My family members and close friends support me to use public transport. | (Dirgahayani and Sutanto, 2020) |
| Perceived accessibility (PA) | PA1: It is easy to do daily activities with public transport.  PA2: If public transport was my only mode of travel, I would be able to continue live the way I want.  PA3: It is possible to do the activities I prefer with public transport.  PA4: Access to my preferred activities is satisfied with public transport. | (Lättman et al., 2016) |
| Intention (IN) | IN1: I intend to reduce car use in the future.  IN2: I intend to use public transport in the future.  IN3: I will use public transport more frequently. | (Chen et al., 2019) |
| Behavior (BE) | BE1: I often travelled by public transport in the last month.  BE2: I used public transport more frequently than driving a car in the last month.  BE3: Public transport was the primary mode for me in the last month. | (Chen et al., 2019) |
