# Supplementary material for: Understanding the determinants for predicting citizens’ travel mode change from private cars to public transport in China
Source: Front Psychol. 2022 Oct 10;13:1007949. doi: 10.3389/fpsyg.2022.1007949 (PMC9588939; doi:10.3389/fpsyg.2022.1007949)
Supplement: Supplementary file 2 [file Table_2.docx]

**Table A2.** Cross-loadings.

| **Constructs** | **AC** | **AR** | **AT** | **BE** | **IN** | **PA** | **PBC** | **PN** | **SN** |
| --- | --- | --- | --- | --- | --- | --- | --- | --- | --- |
| AC1 | **0.850** | 0.331 | 0.122 | 0.246 | 0.269 | 0.228 | 0.203 | 0.453 | 0.183 |
| AC2 | **0.889** | 0.363 | 0.151 | 0.228 | 0.229 | 0.198 | 0.240 | 0.415 | 0.199 |
| AC3 | **0.876** | 0.366 | 0.102 | 0.167 | 0.191 | 0.135 | 0.227 | 0.339 | 0.134 |
| AR1 | 0.323 | **0.882** | 0.208 | 0.156 | 0.266 | 0.247 | 0.132 | 0.374 | 0.227 |
| AR2 | 0.349 | **0.924** | 0.244 | 0.190 | 0.296 | 0.305 | 0.098 | 0.417 | 0.293 |
| AR3 | 0.432 | **0.941** | 0.257 | 0.224 | 0.325 | 0.288 | 0.174 | 0.432 | 0.290 |
| AT1 | 0.059 | 0.180 | **0.905** | 0.527 | 0.637 | 0.640 | 0.295 | 0.371 | 0.595 |
| AT2 | 0.154 | 0.249 | **0.944** | 0.472 | 0.622 | 0.633 | 0.283 | 0.447 | 0.676 |
| AT3 | 0.185 | 0.289 | **0.936** | 0.500 | 0.666 | 0.661 | 0.323 | 0.490 | 0.652 |
| BE1 | 0.220 | 0.185 | 0.526 | **0.960** | 0.634 | 0.627 | 0.311 | 0.378 | 0.469 |
| BE2 | 0.233 | 0.208 | 0.537 | **0.975** | 0.640 | 0.652 | 0.328 | 0.399 | 0.491 |
| BE3 | 0.264 | 0.216 | 0.502 | **0.972** | 0.634 | 0.644 | 0.334 | 0.422 | 0.441 |
| IN1 | 0.284 | 0.322 | 0.599 | 0.562 | **0.916** | 0.686 | 0.402 | 0.661 | 0.606 |
| IN2 | 0.213 | 0.290 | 0.663 | 0.627 | **0.948** | 0.742 | 0.410 | 0.567 | 0.641 |
| IN3 | 0.250 | 0.302 | 0.678 | 0.650 | **0.942** | 0.728 | 0.380 | 0.619 | 0.624 |
| PA1 | 0.171 | 0.253 | 0.663 | 0.563 | 0.690 | **0.852** | 0.258 | 0.408 | 0.594 |
| PA2 | 0.175 | 0.260 | 0.502 | 0.597 | 0.614 | **0.813** | 0.241 | 0.414 | 0.517 |
| PA3 | 0.221 | 0.288 | 0.642 | 0.593 | 0.723 | **0.934** | 0.353 | 0.469 | 0.623 |
| PA4 | 0.194 | 0.278 | 0.639 | 0.581 | 0.680 | **0.924** | 0.306 | 0.448 | 0.608 |
| PBC1 | 0.258 | 0.166 | 0.267 | 0.211 | 0.332 | 0.251 | **0.830** | 0.304 | 0.234 |
| PBC2 | 0.199 | 0.100 | 0.301 | 0.317 | 0.359 | 0.268 | **0.853** | 0.308 | 0.241 |
| PBC3 | 0.188 | 0.109 | 0.239 | 0.302 | 0.367 | 0.303 | **0.813** | 0.248 | 0.242 |
| PN1 | 0.460 | 0.418 | 0.380 | 0.370 | 0.589 | 0.432 | 0.277 | **0.898** | 0.422 |
| PN2 | 0.446 | 0.434 | 0.461 | 0.373 | 0.610 | 0.474 | 0.317 | **0.932** | 0.473 |
| PN3 | 0.361 | 0.366 | 0.447 | 0.387 | 0.599 | 0.443 | 0.348 | **0.905** | 0.452 |
| SN1 | 0.215 | 0.288 | 0.362 | 0.263 | 0.439 | 0.416 | 0.281 | 0.426 | **0.720** |
| SN2 | 0.150 | 0.260 | 0.466 | 0.284 | 0.446 | 0.489 | 0.211 | 0.332 | **0.765** |
| SN3 | 0.160 | 0.212 | 0.656 | 0.470 | 0.620 | 0.611 | 0.241 | 0.443 | **0.859** |
| SN4 | 0.162 | 0.258 | 0.652 | 0.455 | 0.589 | 0.586 | 0.247 | 0.417 | **0.894** |
| SN5 | 0.140 | 0.218 | 0.661 | 0.471 | 0.619 | 0.611 | 0.209 | 0.402 | **0.864** |

Note: The bold values represent each item’s factor loading to its own construct. AC = awareness of consequences, AR = ascription of responsibility, PN = personal norms, PBC = perceived behavioral control, AT = attitudes, SN = subjective norms, PA = perceived accessibility, IN = intention, BE = behavior.
